# Multi-omics profiling and digital image analysis reveal the potential prognostic and immunotherapeutic properties of CD93 in stomach adenocarcinoma

**DOI:** 10.3389/fimmu.2023.984816

**Published:** 2023-01-25

**Authors:** Baokang Wu, Lei Fu, Xingqi Guo, Huixin Hu, Yang Li, Yu Shi, Yizhou Zhang, Shukun Han, Chao Lv, Yu Tian

**Affiliations:** Department of General Surgery, Shengjing Hospital of China Medical University, Shenyang, China

**Keywords:** CD93, stomach adenocarcinoma, multi-omics, digital image analysis, immunotherapy

## Abstract

**Background:**

Recent evidence highlights the fact that immunotherapy has significantly improved patient outcomes. CD93, as a type I transmembrane glycoprotein, was correlated with tumor-associated angiogenesis; however, how CD93 correlates with immunotherapy in stomach adenocarcinoma (STAD) remains unclear.

**Methods:**

TCGA, GTEx, GEO, TIMER2.0, HPA, TISIDB, TCIA, cBioPortal, LinkedOmics, and ImmuCellAI public databases were used to elucidate CD93 in STAD. Visualization and statistical analysis of data were performed by R (Version 4.1.3), GraphPad (Version 8.0.1), and QuPath (Version 0.3.2).

**Results:**

CD93 was highly expressed in STAD compared with adjacent normal tissues. The overexpression of CD93 was significantly correlated with a poor prognosis in STAD. There was a negative correlation between CD93 expression levels with CD93 mutation and methylation in STAD. Our results revealed that CD93 expression was positively associated with most immunosuppressive genes (including PD-1, PD-L1, CTLA-4, and LAG3), immunostimulatory genes, HLA, chemokine, and chemokine receptor proteins in STAD. Furthermore, in STAD, CD93 was noticeably associated with the abundance of multiple immune cell infiltration levels. Functional HALLMARK and KEGG term enhancement analysis of CD93 through Gene Set Enrichment Analysis was correlated with the process of the angiogenesis pathway. Subsequently, digital image analysis results by QuPath revealed that the properties of CD93^+^ cells were statistically significant in different regions of stomach cancer and normal stomach tissue. Finally, we utilized external databases, including GEO, TISIDB, ImmuCellAI, and TCIA, to validate that CD93 plays a key role in the immunotherapy of STAD.

**Conclusion:**

Our study reveals that CD93 is a potential oncogene and is an indicative biomarker of a worse prognosis and exerts its immunomodulatory properties and potential possibilities for immunotherapy in STAD.

## Introduction

CD93, as a type I transmembrane glycoprotein with one C-type lectin-like domain, five tandem EGF-like domain repeats, a serine threonine-rich mucin-like domain, a transmembrane domain, and a cytoplasmic domain (1, 2), is prominently expressed in endothelial cells and certain hematopoietic subsets (2). It was involved in the process of angiogenesis (3). The latest research revealed that CD93 was also highly expressed in tumor-associated vasculature, including nasopharyngeal carcinoma, glioblastoma, colorectal cancer, and pancreatic ductal adenocarcinoma (4–7). According to GLOBOCAN 2020, approximately 1,089,103 new cases and 768,793 deaths occur in 2020 and are associated with stomach cancer, making it the fifth most common cancer (excluding non-melanoma skin cancer) and the third most common cause of death by cancer among 36 cancer types, thus contributing to the high burden all around the world (8). With the advent of immune checkpoint blockades (ICBs), such as PD-1, PD-L1, and CTLA-4 monoclonal antibodies (mAbs), these offer novel treatment possibilities for solid cancer, with the crucial benefit of providing higher cure rates (9). Increasing evidence demonstrated that the application of ICBs is a new standard of targeted therapy in the treatment of stomach cancer and other kinds of cancers (10). However, there is still a significant proportion of patients who showed minimal response to ICBs. Recently, Sun and colleagues demonstrated that CD93 interacting with its receptor insulin-like growth factor binding protein 7 (IGFBP7) could contribute to abnormal tumor vasculature in human umbilical vein endothelial cells and influence tumor growth in *in vitro* murine KPC model (7). The blockade of the CD93 pathway by mAbs promoted vascular maturation, leading to an improved antitumor response to gemcitabine or fluorouracil (7). Furthermore, the blockade of CD93 pathway increased immune T cell infiltration and antitumor immunity, resulting in sensitizing tumors to ICB therapy (7). This study identified CD93 to be involved in tumor vascular dysfunction and revealed an approach to promote a favorable tumor immune microenvironment for therapeutic intervention. Meanwhile, Zhu and colleagues revealed that CD93 was associated with immune subtypes characterized by a distinct tumor immune microenvironment and patient prognosis in stomach adenocarcinoma (STAD) (11).

In this study, a comprehensive analysis of the CD93 function in STAD was carried out using multi-omics databases. We explored the CD93 expression level and survival analysis in STAD. The results showed that it was highly expressed and could serve as a diagnostic and prognostic biomarker in STAD. Subsequently, we further explored the correlation of CD93 expression and mutation and methylation. In addition, we analyzed the connection between CD93 expression and immune-related genes, immune infiltrates in the tumor microenvironment (TME), and enrichment function analysis. Moreover, we checked for CD93 expression difference between stomach cancer and normal stomach tissue by using the digital image analysis (DIA) software QuPath. We validated the correlation between CD93 and immunity, prognosis, and immunotherapy with external databases.

## Materials and methods

### Data source and processing

The RNA sequence and related clinical data of STAD were acquired from The Cancer Genome Atlas (TCGA), and the normal human tissue profile data were retrieved from Genotype Tissue Expression (GTEx) through the UCSC cancer genome browser (https://xena.ucsc.edu/). Normal sample transcriptome RNA-seq data from both TCGA and GTEx databases were used for comparisons between cancer and normal tissue; we dealt with the batch effect with the “limma” and “sva” packages in R.

### Analysis of mutation and methylation in CD93

CD93 genomic (mutation and copy number variation, CNV) and epigenomic (methylation) analysis in STAD was determined using the cBioPortal for Cancer Genomics (http://www.cbioportal.org/) platform. The methylation data of CD93 was collected from the LinkedOmics database (http://www.linkedomics.org/login.php), and the association between CD93 expression and methylation was calculated by Spearman’s correlation coefficients.

### Analysis of CD93 in TME

In the first method, the TIMER2.0 database (http://timer.cistrome.org/) was examined to elucidate the association of CD93 level with immune-related genes, including immunosuppressive genes, immunostimulatory genes, HLA, chemokine, and chemokine receptor proteins, through calculating the Spearman’s correlation coefficients in STAD. Notably, we investigated the correlation of CD93 expression with PD-1, PD-L1, LAG3, and CTLA-4 levels by calculating the Spearman’s correlation coefficients through the TISIDB database (http://cis.hku.hk/TISIDB/). In the second method, we surveyed the relationship of CD93 expression with TME *via* evaluating the ratio of stromal and immune cells in tumors by “estimate” packages in R, and the quantified results were displayed as stromal score, immune score, and ESTIMATE score in STAD. In the third approach, we next explored the relationship between CD93 expression and tumor purity and tumor-infiltrating immune cells in STAD by calculating the Spearman’s correlation coefficients through the TIMER2.0 database.

### Gene set enrichment analysis

Gene Set Enrichment Analysis (GSEA) was performed to investigate the differential pathways [HALLMARK and Kyoto Encyclopedia of Genes and Genomes (KEGG)] among the low- and high-CD93-expression groups in STAD (according to the median, the data was divided into two types: low- and high-CD93-expression groups). The HALLMARK gene sets and KEGG gene sets were downloaded from the official GSEA website (https://www.gsea-msigdb.org/gsea/downloads.jsp). The enriched gene sets were selected based on the value of normalized enrichment score (NES), false discovery rate (FDR), and *p*-value; *p <*0.05 and FDR ≤0.25 were considered to indicate statistical significance.

### Pathology image and validation by external databases

Immunohistochemical (IHC) images of stomach cancer stained by CD93 antibody were collected from the Human Protein Atlas (HPA) (https://www.proteinatlas.org/) database. The IHC inclusion criteria were as follows: (1) the staining image was clear and (2) the results of tissue and cell partition analyzed by DIA were generally consistent with the pathology results. The IHC exclusion criterion was as follows: the tissue residue in the IHC image is less than half the normal tissue.

We downloaded transcriptome profiles and related clinical data of STAD (GSE26253, *n* = 432) from the Gene Expression Omnibus (GEO; https://www.ncbi.nlm.nih.gov/geo/) database. Immunotherapy data from the TISIDB database and the immunophenotype (IPS) score data of STAD from the TCIA (https://tcia.at/home) database (a scoring system for predicting the response effects of PD-1 inhibitors and CTLA-4 inhibitors) were used to verify the predictive value of CD93 in immunotherapy response. Furthermore, we acquired the ICB therapy response prediction results by analyzing the GSE26253 (*n* = 432) dataset from the ImmuCellAI (http://bioinfo.life.hust.edu.cn/web/ImmuCellAI/) database.

### Statistical analysis

Univariate and multivariate Cox models were applied to explore the prognostic significance of CD93 in STAD; the results were displayed as hazard ratio (HR), log-rank *p*-values (*p* < 0.05 as significant), and 95% confidence intervals (95% CI). Furthermore, we downloaded one outcome parameter overall survival (OS) data from TCGA. We generated a Kaplan–Meier survivorship curve to compare the OS rate for patients based on CD93 expression using the log-rank test (*p <*0.05 as significant). The above-mentioned results were visualized with the “forestplot” and “survival” packages in R.

The Shapiro–Wilk test was used for the normality test. Student’s *t*-test (meet the normal distribution) and Mann–Whitney *U*-tests (do not meet the normal distribution) were used for comparisons between the two groups’ data from DIA analysis. Spearman’s correlation analysis was used to measure the degree of correlation between certain variables, and the following R/rho values were used to judge the correlations: 0–0.19, “very weak”; 0.20–0.39, “weak”; 0.40–0.59, “moderate”; 0.60–0.79, “strong”; 0.80–1.00, “very strong”; *p <*0.05 was considered significant. All statistical analyses were processed by the R (Version 4.1.3) and GraphPad (Version 8.0.1) software. IHC images were analyzed by QuPath (Version 0.3.2).

## Results

### The expression landscape of CD93 in STAD

**Figure 1:**
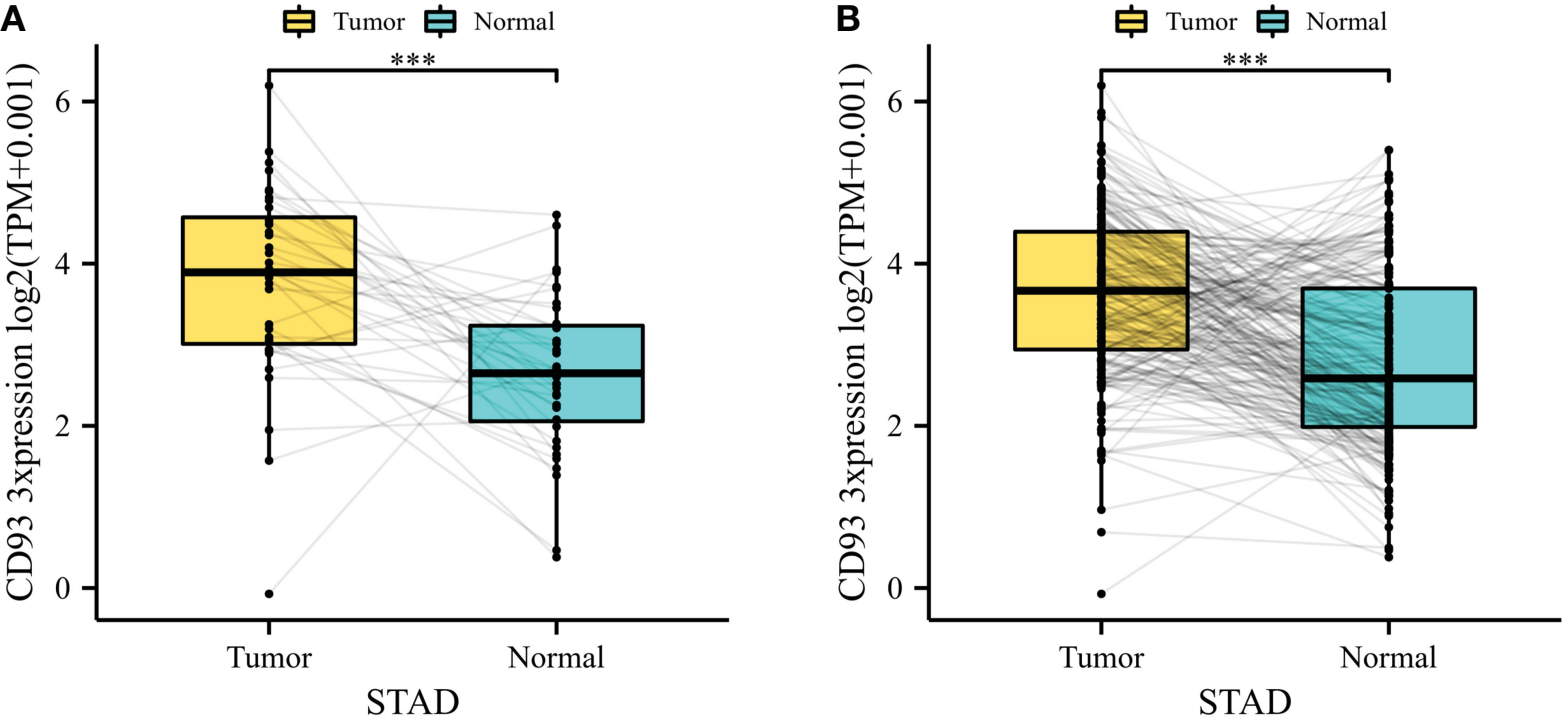
**(A)** CD93 expression difference between stomach adenocarcinoma (STAD) and adjacent normal tissue in The Cancer Genome Atlas (TCGA) database. **(B)** CD93 expression difference between STAD and adjacent normal tissue in the integrated database of TCGA and GTEx. ***p < 0.001.

To explore the basic expression of CD93, we first analyzed the mRNA expression of CD93 expression levels between normal and tumor samples in datasets from TCGA database which revealed that a significantly increased expression was found in STAD (**Figure 1A**). In the combined datasets based on an integrated database of GTEx and TCGA datasets, CD93 expression was significantly upregulated in the tumor samples of STAD (**Figure 1B**). The results showed that CD93 was remarkably upregulated in STAD than in normal samples (*p* < 0.001).

### CD93 mutation analysis and methylation analysis

**Figure 2:**
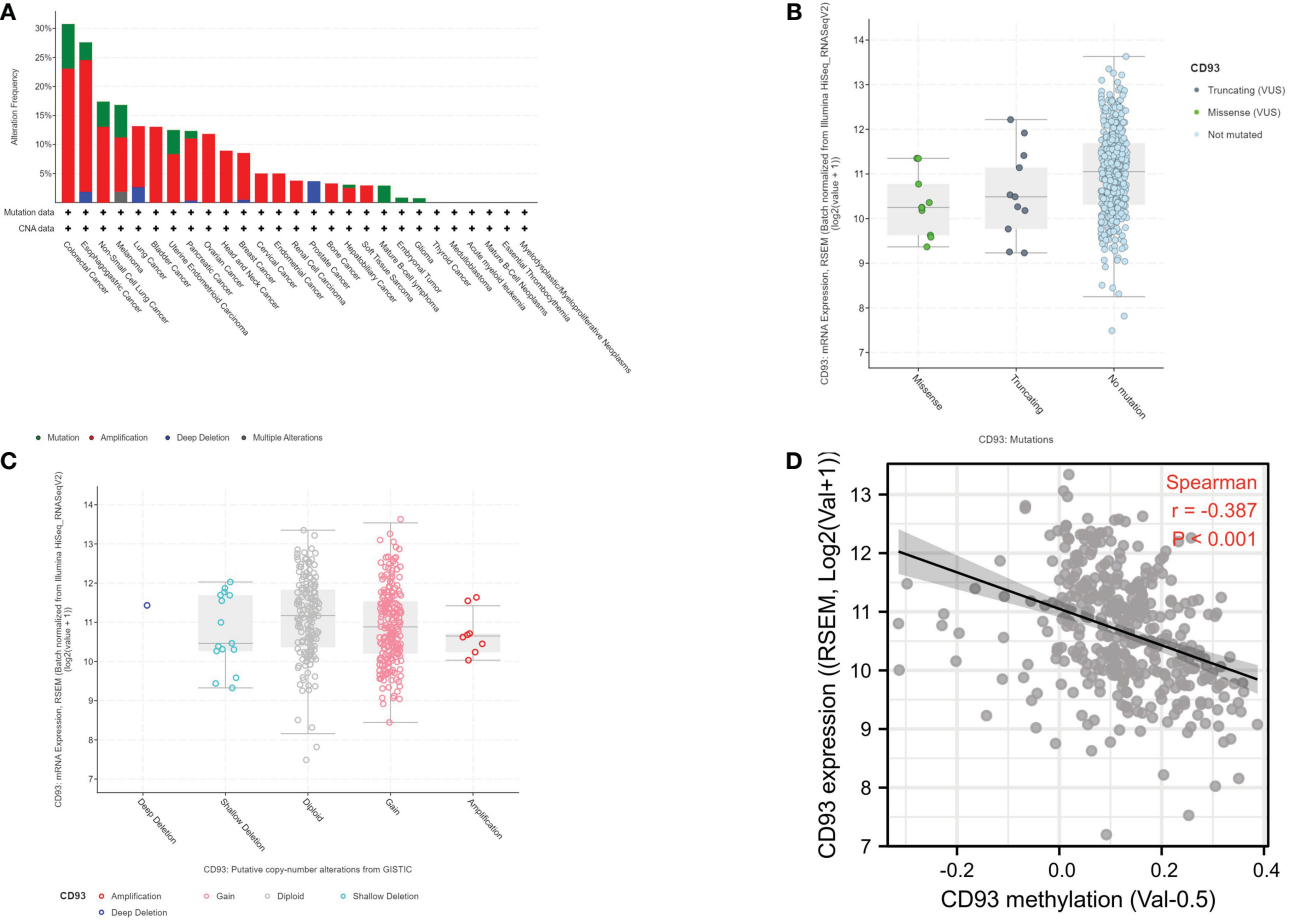
**(A)** CD93 mutation frequency in stomach adenocarcinoma (STAD) according to the cBioportal database. **(B)** CD93 expression level in different mutation types in STAD according to the cBioportal database. **(C)** CD93 expression level in different copy number variation types in STAD according to the cBioportal database. **(D)** Correlation between CD93 expression and CD93 methylation in STAD (Spearman, *r* = -0.387, *p* < 0.001).

It is widely known that genomic and epigenomic changes play a key role in the regulation of gene expression and TME, which influence the development and progression of cancer (12–14). Firstly, we analyzed the alteration frequency of CD93 in STAD in the cBioportal database (**Figure 2A**). The results showed that the alteration frequency of CD93 was the second highest in esophagogastric cancer, and the main type is the amplification mutation. Secondly, we analyzed the correlation between CD93 mutation and CD93 mRNA expression in STAD (*n* = 407) (**Figure 2B**). The results showed that the STAD samples with no mutation have the highest average expression, followed by the second and third average expressions which were truncating and missense, respectively. Subsequently, we investigated the correlation between CD93 CNV and CD93 mRNA expression in STAD (*n* = 407) (**Figure 2C**). The results revealed that the type of deep deletion has the highest average expression, followed by the type of diploid, gain, amplification, and shallow deletion, respectively. Finally, we explored the correlation between CD93 methylation and CD93 mRNA expression in STAD in LinkedOmics database. The data demonstrated a strongly negative correlation (Spearman: *r* = -0.378, *p* < 0.001).

### Correlation of CD93 expression and prognostic value in STAD

**Figure 3:**
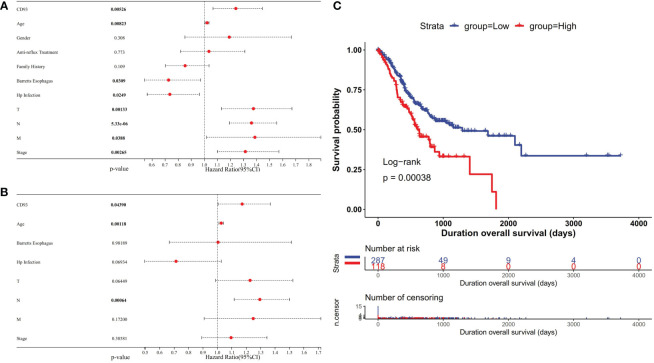
Forest plot showing the univariate **(A)** and multivariate **(B)** Cox regression analysis results in stomach adenocarcinoma (STAD). **(C)** Kaplan–Meier curves showing that the high CD93 expression was correlated with poor prognosis in STAD.

Usually, differential expression genes are associated with patient survival and prognosis. From the above-mentioned results, it was well known that CD93 was differently expressed between certain tumors and normal tissues. To understand the correlation of CD93 expression level and prognosis, we used univariate and multivariate Cox regression models to evaluate the OS in STAD. The results showed that high CD93 expression was an independent unfavorable prognostic gene in STAD [univariate analysis: *p* = 0.00526 (**Figure 3A**), multivariate analysis: *p* = 0.04390 (**Figure 3B**)]. Furthermore, we evaluated similar results by Kaplan–Meier survival analysis (**Figure 3C**); CD93 was found as an unfavorable factor in STAD (*p* = 0.00038).

### Correlation between CD93 and immune-related genes, and immune infiltrates

**Figure 4:**
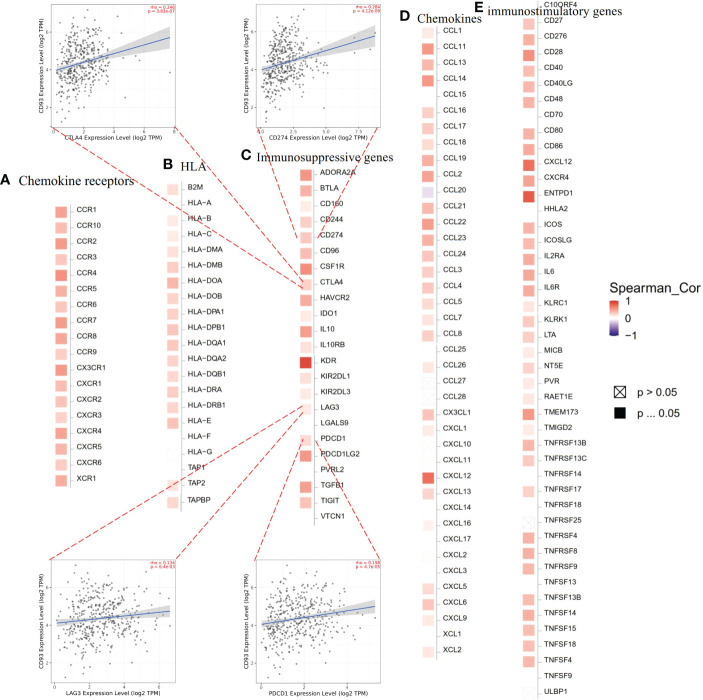
Expression correlations between CD93 and immune-related genes according to TIMER2.0 database: **(A)** chemokine receptor genes, **(B)** HLA, **(C)** immunosuppressive genes, **(D)** chemokine genes, and **(E)** immunostimulatory genes in stomach adenocarcinoma.

The TME is known to play a vital role in regulating malignant tumors’ progress and modulating reactions to therapies. Recently, therapies targeting the TME have emerged as a promising method for cancer treatment (15). As important components of the TME, immune cells and immune-related genes make a great contribution to the homeostasis and evolution of the TME. To understand the association between CD93 and immune-related genes including immunosuppressive genes, immunostimulatory genes, HLA, chemokines, and chemokine receptor genes, we conducted CD93 and immune-related gene co-expression analyses in STAD from TCGA in the TIMER2.0 database. The results revealed that CD93 exhibited a positive association with most immunosuppressive genes, immunostimulatory genes, HLA, chemokines, and chemokine receptor genes in STAD (**Figures 4A–E**). In STAD, we found that there was the strongest correlation between CD93 expression and the expression levels of recognized immune checkpoints including KDR in immunosuppressive genes (**Figure 4C**), ENTPD1 in immunostimulatory genes (**Figure 4E**), HLA-DOA in HLA genes (**Figure 4B**), CXCL12 in chemokine genes (**Figure 4D**), and CCR4 in chemokine receptor genes (**Figure 4A**). Furthermore, we investigated the co-expression correlation between CD93 and several of the most common immune checkpoint molecules focused on by researchers, such as PD-1 (PDCD1), PD-L1 (CD274), CTLA-4, and LAG3. The results showed a strong correlation (PD-1: Spearman rho = 0.198, *p* = 4.7e-05; PD-L1: Spearman rho = 0.284, *p* = 4.12e-09; CTLA-4: Spearman rho = 3.83e-07; LAG3: Spearman rho = 0.134, *p* = 6.4e-03) (**Figure 4C**).

**Figure 5:**
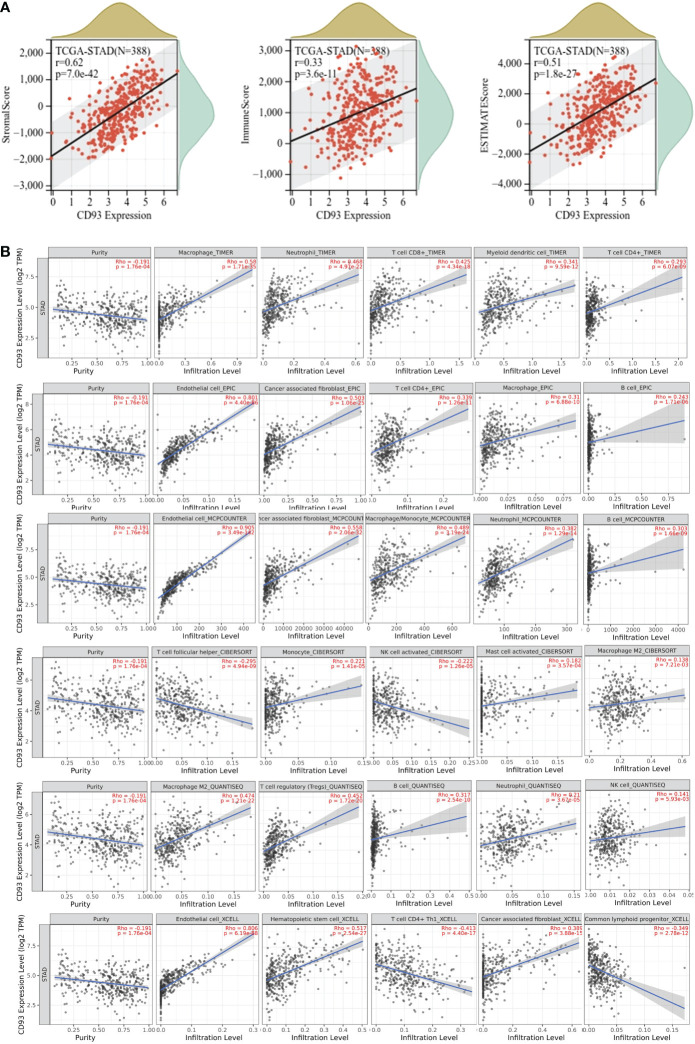
**(A)** Correlation of CD93 expression with stromal score (Spearman, *r* = 0.62, *p* = 8.3e-43), immune score (Spearman, *r* = 0.34, *p* = 3.2e-12), and ESTIMATE score (Spearman, *r* = 0.52, *p* = 2.4e-28) in stomach adenocarcinoma (STAD). **(B)** Correlations of CD93 expression and immune cell infiltration level in STAD.

Next, we explored the correlation of CD93 expression and TME. We calculated the stromal score, immune score, and ESTIMATE score in STAD (**Figure 5A**). Our results indicated a strongly positive correlation among them. Moreover, we calculated the tumor purity and several infiltrating immune cells in the TIMER2.0 database. Our analysis indicated a negative relationship between CD93 expression and tumor purity in STAD (Spearman rho = -0.191, *p* = 1.76e-04) (**Figure 5B**). Furthermore, we assessed the correlation of CD93 expression and the top five infiltrating immune cells in STAD using TIMER, EPIC, MCPCOUNTER, CIBERSORT, QUANTISEQ, and xCELL algorithms (**Figure 5B**). The results revealed that the top five infiltrating immune cells were macrophage (Spearman rho = 0.58, *p* = 1.71e-35), neutrophil (Spearman rho = 0.468, *p* = 4.97e-22), T cell CD8+ (Spearman rho = 0.425, *p* = 4.34e-18), myeloid dendritic cell (Spearman rho = 0.341, *p* = 9.59e-12), and T cell CD4+ (Spearman rho = 0.293, *p* = 6.07e-09) in TIMER; endothelial cell (Spearman rho = 0.801, *p* = 4.40e-86), cancer-associated fibroblast (Spearman rho = 0.503, *p* = 1.06e-25), T cell CD4+ (Spearman rho = 0.339, *p* = 1.26e-11), macrophage (Spearman rho = 0.31, *p* = 6.88e-10), and B cell (Spearman rho = 0.243, *p* = 1.71e-06) in ERIC; endothelial cell (Spearman rho = 0.905, *p* = 3.49e-142), cancer-associated fibroblast (Spearman rho = 0.558, *p* = 2.06e-32), macrophage/monocyte (Spearman rho = 0.489, *p* = 3.19e-24), neutrophil (Spearman rho = 0.382, *p* = 1.29e-14), and B cell (Spearman rho = 0.303, *p* = 1.66e-09) in MCPCOUNTER; T cell follicular helper (Spearman rho = -0.295, *p* = 4.94e-09), monocyte (Spearman rho = 0.221, *p* = 1.1e-05), NK cell activated (Spearman rho = -0.202, *p* = 1.26e-05), mast cell activated (Spearman rho = 0.182, *p* = 3.57e-04), and macrophage M2 (Spearman rho = 0.138, *p* = 7.21e-03) in CIBERSORT; macrophage M2 (Spearman rho = 0.474, *p* = 1.21e-22), T cell regulatory (Tregs) (Spearman rho = 0.452, *p* = 1.72e-20), B cell (Spearman rho = 0.317, *p* = 2.54e-10), neutrophil (Spearman rho = 0.21, *p* = 3.67e-05), and NK cell (Spearman rho = 0.141, *p* = 5.93e-03) in QUANTISEQ; and endothelial cell (Spearman rho = 0.806, *p* = 6.19e-88), hematopoietic cell (Spearman rho = 0.517, *p* = 2.54e-27), T cell CD4+ Th1 (Spearman rho = -0.413, *p* = 4.40e-17), cancer-associated fibroblast (Spearman rho = 0.389, *p* = 3.88e-15), and common lymphoid progenitor (Spearman rho = -0.349, *p* = 2.78e-12) in xCELL.

### Functional analysis by GSEA

**Figure 6:**
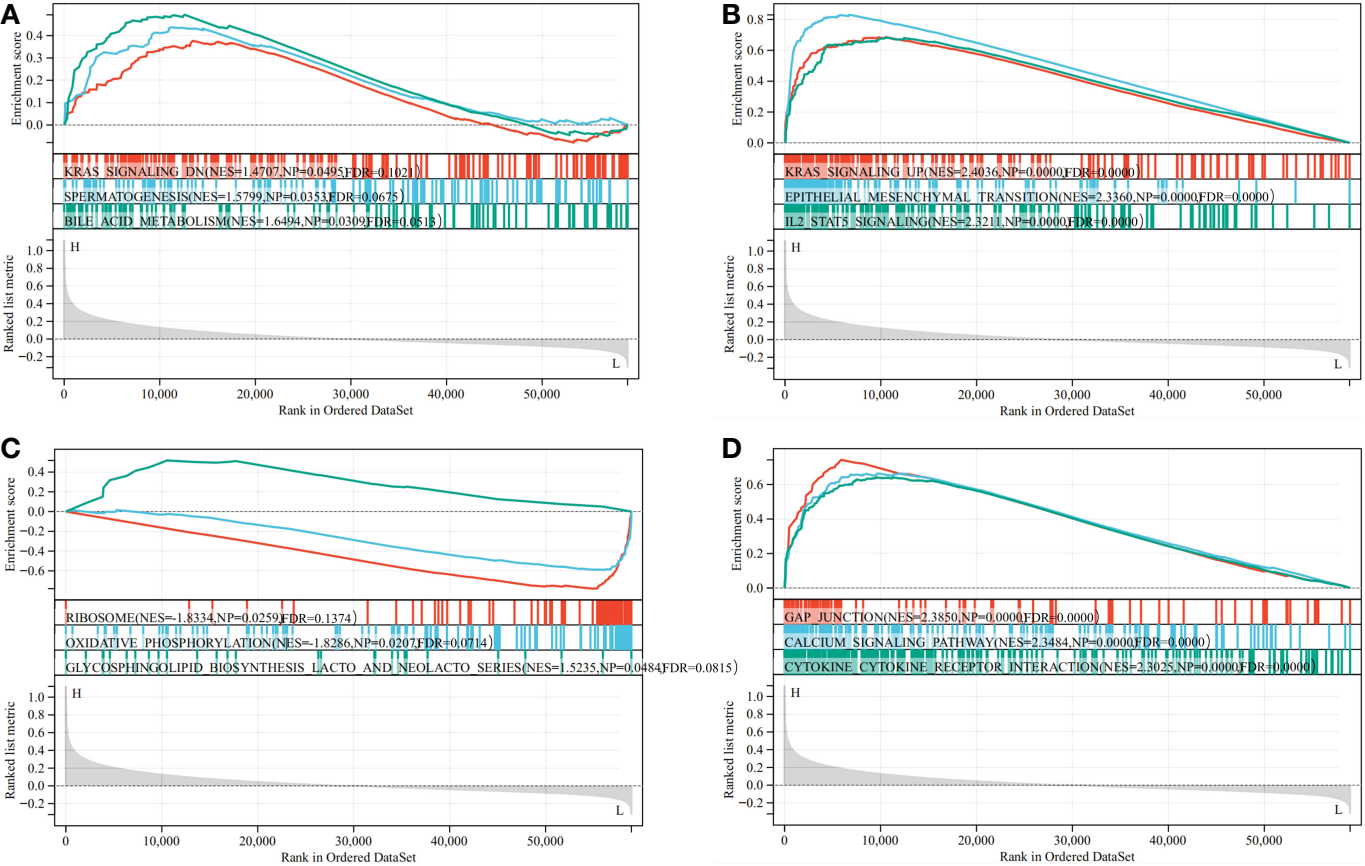
Result of Gene Set Enrichment Analysis (GSEA). GSEA (HALLMARK **(A, B)** and Kyoto Encyclopedia of Genes and Genomes **(C, D)** between CD93 high- and low-expression groups in stomach adenocarcinoma.

The biological role of CD93 in STAD was illustrated through GSEA. Functional HALLMARK and KEGG terms of CD93 were analyzed. The results revealed that the top three HALLMARK terms with the lowest value of NES in the high-CD93 group were Kras-signaling-DN, spermatogenesis, and bile-acid-metabolism (**Figure 6A**), and the top three HALLMARK terms with the highest value of NES were kras-signaling-UP, epithelial-mesenchymal-transition, and IL2-STAT5-signaling (**Figure 6B**). The top three KEGG terms with the lowest value of NES in the high-CD93 group were ribosome, oxidative-phosphorylation, and glycosphingolipid-biosynthesis-lacto-and-neolacto-series (**Figure 6C**), and the top three KEGG terms with the highest value of NES were gap-junction, calcium-signaling-pathway, and cytokine-cytokine-receptor interaction (**Figure 6D**).

### CD93 expression analyzed by DIA

**Figure 7:**
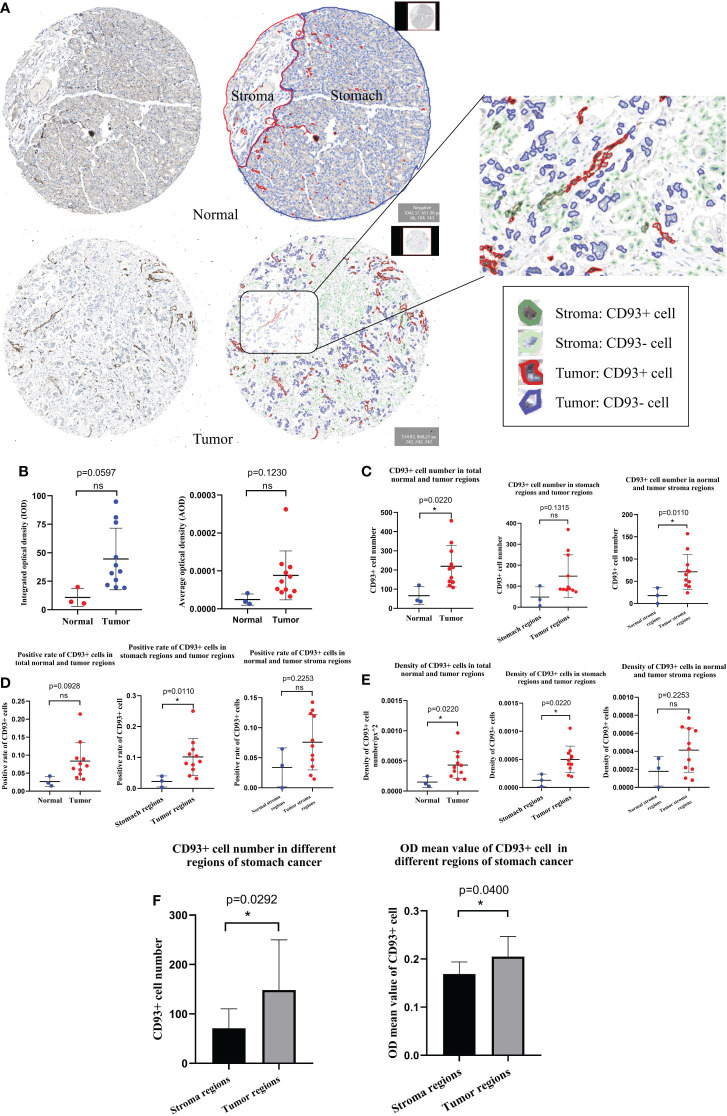
Human Protein Atlas immunohistochemical staining image analysis results by QuPath. **(A)** Results of tissue segmentation and cell recognition of stomach cancer and normal stomach tissue. **(B)** The integral optical density and average optical density of CD93^+^ cells in stomach cancer and normal stomach tissue were statistically significant. The CD93^+^ cell number **(C)**, positive rate **(D)**, and density **(E)** were statistically significant in different regions of stomach cancer and normal stomach tissue. **(F)** Contrast results of CD93^+^ cell number and optical density values of CD93^+^ cells in different regions of stomach cancer. Student’s *t*-test (meet the normal distribution) and Mann–Whitney *U*-tests (do not meet the normal distribution). *p < 0.05. ns, no significance.

Digital images analysis technology is convenient for image analysis. It is widely applied in diagnostic laboratories, translational research, and drug development. We qualitatively and quantitatively performed IHC staining images from the HPA database with the DIA software QuPath (**Figure 7A**). Combined with the results of the region of interest drawn by the pathologist, we recognized the different regions in stomach cancer (tumor and stroma) and normal stomach tissue (stomach and stroma) IHC staining images by QuPath. Furthermore, we recognized the CD93^+^ and CD93^-^ cells in different regions of stomach cancer and normal stomach tissue. The integral optical density (IOD) and the average optical density (AOD) of stomach cancer and normal stomach tissue were obtained by semi-quantitative analysis. The results revealed that the difference was not significant (IOD: *p* = 0.0597, AOD: *p* = 0.1230) (**Figure 7B**). Based on the results of tissue segmentation and cell recognition, we observed that the number of CD93^+^ cells in the whole visual field of stomach cancer was more than in normal stomach tissue (*p* = 0.0220); the number of CD93^+^ cells in different stroma regions were statistically significant (*p* = 0.0110) (**Figure 7C**). The positive rate of CD93^+^ cells in tumor regions was higher than in stomach regions (*p* = 0.0110) (**Figure 7D**). Beyond that, the density of CD93^+^ cells in tumor regions was higher than in stomach regions (*p* = 0.0220). The density of CD93^+^ cells in tumor regions was higher than in stomach regions (*p* = 0.0220) (**Figure 7E**). Next, we determined that the CD93^+^ cell number and the OD values of CD93^+^ cells in different regions of stomach cancer were significantly different (*p* = 0.0292 and *p* = 0.0400, respectively) (**Figure 7F**).

### External datasets validate the correlation between CD93 gene and immunity, prognosis, and immunotherapy

**Figure 8:**
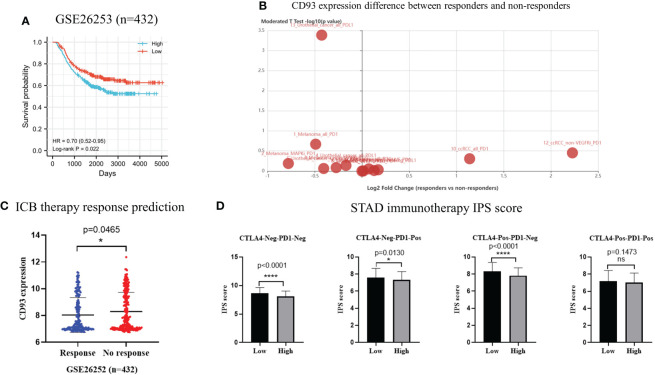
**(A)** Kaplan–Meier curves showing overall survival in 432 stomach adenocarcinoma patients from GEO database (GSE26253) [log-rank test, HR = 0.70 (0.52–0.95), *p* = 0.0220]. **(B)** CD93 expression difference between immunotherapy responders and non-responders according to TISIDB database. **(C)** Predicted results of immune checkpoint blockade therapy response based on ImmuCellAI database. **(D)** The immunophenotype scores in low-CD93 group and high-CD93 group. *p < 0.05, ****p < 0.0001. ns, no significance.

To verify the prognostic significance of CD93 in stomach cancer, we downloaded transcriptome and related clinical data from GEO datasets (GSE26253, *n* = 432). According to Kaplan–Meier survival curve, patients in the high-CD93 group lived longer, and the difference was statistically significant [log-rank test, *P* = 0.022, HR = 0.70 (0.52–0.95)] (**Figure 8A**). CD93 expression was different in the immunotherapy cohort and non-response cohort in the TISIDB database (**Figure 8B**). We predicted ICB therapy response with the data from GSE26253 in the ImmuCellAI database; the CD93 expression was significantly decreased in the response cohort compared with the no-response cohort (*p* = 0.0465) (**Figure 8C**). We also obtained IPS scores for immunotherapeutic response from the TCIA database that predicted the efficacy of PD-1 and CTLA4 immune checkpoint blockades and found that the IPS scores of the low-CD93-expression group was significantly higher than those of the high-CD93-expression group in CTLA4-Neg-PD1-Neg, CTLA4-Neg-PD1-Pos, and CTLA4-Pos-PD1-Neg cohorts (*p* < 0.0001, *p* = 0.0130, and *p* < 0.0001, respectively) (**Figure 8D**). The results showed that the immunotherapy efficacy of PD-1 inhibitors or CTLA4 inhibitors could be enhanced when combined with anti-CD93 therapy in STAD.

## Discussion

Through high-throughput bioinformatics analysis, the present work illustrated a comprehensive workflow for STAD and thoroughly elucidated the role of CD93 in cancer. Analysis based on TCGA and GTEx data showed that CD93 expression was significantly increased in STAD compared with normal tissues. Meanwhile, the survival analysis based on univariate and multivariate Cox regression analysis showed that CD93 was an independent prognostic factor. Overexpression of CD93 indicated a poor prognosis in STAD. Supporting that is the fact that increasing evidence had shown that CD93 plays an important role in the angiogenesis and vasculature of cancer, including nasopharyngeal carcinoma, glioblastoma, colorectal cancer, and pancreatic ductal adenocarcinoma (4–7). In addition, CD93 was found to be correlated with the prognosis of non-cancer diseases. CD93 signaling is a leukemia stem cell-specific regulator of self-renewal and proliferation and a targetable pathway to eliminate leukemia stem cells in chronic myeloid leukemia (16). CD93 chimeric antigen receptor T cells eliminate acute myeloid leukemia and spare hematopoietic stem and progenitor cells but exert on-target, off-tumor toxicity to endothelial cells (17). Notably, the expression of CD93 has an essential role in the pathogenesis of psoriasis, cardiovascular, and cerebrovascular diseases (18–20). These findings provide an idea for targeting CD93 therapy. Based on bioinformatics analysis, CD93 was significantly highly expressed in STAD than in adjacent normal tissues, and the overexpression of CD93 was correlated with a poor prognosis in STAD. However, basic studies focused on investigating the underlying molecular mechanism of the relationship between CD93 and STAD are relatively scarce.

In our study, we analyzed the relationship between CD93 expression and its mutation and methylation. In the cBioportal database, we showed the alteration frequency of CD93 in STAD. In addition, we analyzed the correlation between CD93 mutation and CD93 mRNA expression in STAD. The results showed a negative correlation between CD93 mutation and CD93 expression in STAD. Subsequently, we investigated the correlation between CD93 CNV and CD93 mRNA expression in STAD. Finally, we explored the correlation between CD93 methylation and CD93 mRNA expression in STAD in the LinkedOmics database. These findings suggested that CD93 mutation and methylation decreased the CD93 mRNA expression level, resulting in altering the function of CD93.

The TME is an environment conducive to the growth and expansion of cancer cells, which comprise a variety of immune cells, including helper T cells, regulatory T cells, dendritic cells, tumor-associated macrophages, and mesenchymal stem cells in stomach cancer (21). There is an increasing number of studies which found that targeted TME therapy has been involved in the treatment of stomach cancer. To further elucidate the underlying mechanism of the relationship between CD93 and stomach cancer, we analyzed the relationship between the expression of CD93 and five immune modes (including immunosuppressive genes, immunostimulatory genes, HLA, chemokines, and chemokine receptor genes) in 33 types of cancer. The results revealed that CD93 shows a strong correlation with the most immune mode genes. Particularly, we show the relationship between CD93 and PD-1, PD-L1, CTLA-4, and LAG3 in STAD. In the TME, stromal and immune cells had been considered to play a crucial role to maintain the stability of homeostasis (22). In tumors, stromal and immune scores reflect the strength of immunity. Our results revealed that CD93 shows a great correlation with the stromal score, immune score, and ESTIMATE score. Moreover, we found that CD93 was significantly correlated with the immune cell infiltration levels. Previous several studies demonstrated that CD93 was correlated with the abundance of immune cells, including endothelial cells (23), group 3 innate lymphoid cells (24), hematopoietic stem cells and multipotent progenitor cells (25), and T cells (7). Consistently, in STAD, we analyzed that CD93 shows a great correlation with macrophage/monocyte, neutrophil, T cell CD8+, myeloid dendritic cell, T cell CD4+, endothelial cell, cancer-associated fibroblast, B cell, T cell follicular helper, NK cell activated, mast cell activated, macrophage M2, T cell regulatory, NK cell, hematopoietic cell, T cell CD4+ Th1, and common lymphoid progenitor by using TIMER, EPIC, MCPCOUNTER, CIBERSORT, QUANTISEQ, and xCELL algorithms. These findings and our results revealed that CD93 may be used as a potential immunotherapy target by regulating the immune cell infiltration levels in the TME.

Recently, the findings pointed to focusing on CD93 function. Previous studies had demonstrated that CD93 was involved in apoptosis, inflammation, cell adhesion, and angiogenesis (20, 23, 26, 27). To specifically address the function of CD93 in STAD, we performed enrichment analysis (HALLMARK and KEGG) on CD93. We found a strong link between it and Kras-signaling-DN, spermatogenesis, bile-acid-metabolism, Kras-signaling-UP, epithelial-mesenchymal-transition, IL2-STAT5-signaling, ribosome, oxidative-phosphorylation, glycosphingolipid-biosynthesis-lacto-and-neolacto-series, gap-junction, calcium-signaling-pathway, and cytokine-cytokine-receptor interaction.

In this study, we elucidated the expression characteristics of CD93^+^ of STAD IHC staining images from the HPA database analyzed by DIA software QuPath. Based on the results of tissue segmentation and cell recognition, we observed that CD93^+^ cell was overexpressed in stomach cancer compared with normal stomach tissue. According to the qualitative and quantitative analysis results, we found that qualitative and quantitative analyses based on artificial intelligence principles are better than traditional analysis and have a more reliable conclusion (28, 29). The analysis pattern identified IHC staining image features that cannot be observed by the naked eye (30). Notably, we identified multiple pathological features of stomach cancer with the aid of DIA techniques combined with public databases. The innovative combination validated the expression of CD93 in stomach cancer.

In this study, we further analyzed the relationship between CD93 and immunotherapy in STAD and other tumors. Based on the TISIDB database, we observed CD93 expression differences between ICB therapy responders and ICB therapy non-responders of melanoma, urothelial cancer, and clear cell renal cell carcinoma. We predicted the STAD patients’ CD93 expression difference in the ICB therapy response cohort and the ICB therapy no-response cohort. Additionally, based on the IPS scores, the immunotherapy efficacy of PD-1 inhibitors or CTLA4 inhibitors could be enhanced when combined with anti-CD93 therapy in STAD. Consistently, recent research demonstrated that a combination of CD93 and PD-1 mAbs profoundly inhibited pancreatic ductal adenocarcinoma tumor growth in mice (7). Similarly, in the B16 melanoma mouse model, the inclusion of anti-CD93 improved the antitumor effect mediated by PD-1 and CTLA4 mAbs, as revealed by tumor growth and mouse survival curves (7). The researcher also validated that the CD93 pathway in the TME may contribute to cancer resistance to anti-PD therapy in humans (7).

Overall, we analyzed the correlation between CD93 expression and prognosis, immune checkpoints, TME, immune cell infiltration levels, immune-related genes, functional enrichment, and immunotherapy in STAD. More importantly, our study firstly utilized DIA techniques based on artificial intelligence principles combined with public databases to verify the relationship between CD93 and STAD. Our finding suggested that CD93 may be a promising target for immunotherapy of STAD or other types of cancer. However, there are also limitations in our study. We lack further experiments to verify the precise molecular function of CD93 in stomach cancer.

We identified the value of CD93 in STAD by multiple analyses. CD93, as a promising prognostic biomarker, exerts its immunoregulation properties and potential possibilities for tumor immunotherapy in STAD. Additionally, considering that CD93 has been implicated to play roles in the etiology of multiple diseases and therefore multiple pathways that have implications outside of cancer, this makes CD93 a unique and enticing target. Thus, the therapeutic benefit of CD93 is such that of one target with multiple indications.

## Data availability statement

The datasets presented in this study can be found in online repositories. The names of the repository/repositories and accession(s) can be found in the article/supplementary material.

## Author contributions

BW, LF, and XG drafted the manuscript. HH, YL, YS, SH and CL completed the figures. YT managed the article design, reviewed the manuscript, and provided funding support. All authors contributed to the article and approved the submitted version.
